# Chemopreventive Potential of Paddy Waste: A Promising Approach Against Benign Prostate Hyperplasia in Spontaneously Hypertensive Rats

**DOI:** 10.1155/ancp/4029625

**Published:** 2025-03-13

**Authors:** Azman Seeni, Atif Amin Baig, Mogana Das Murtey

**Affiliations:** ^1^Department of Toxicology, Advanced Medical and Dental Institute, Universiti Sains Malaysia, Bertam, Kepala Batas 13200, Penang, Malaysia; ^2^Advanced Material Research Center, SIRIM Industrial Research, SIRIM Berhad, Kulim Hi-Tech Park, Kulim 09000, Kedah, Malaysia; ^3^International Medical School, Management and Science University, University Drive, Off Persiaran Olahraga, Section 13, Shah Alam 40100, Selangor Darul Ehsan, Malaysia; ^4^School of Dental Sciences, Health Campus, Universiti Sains Malaysia, Kubang Kerian 16150, Kelantan, Malaysia

**Keywords:** benign prostate hyperplasia, chemoprevention, husk, paddy waste, prostate

## Abstract

**Background:** Benign prostate hyperplasia (BPH) is common in elderly men. Previously, paddy waste (both husk and straw) reportedly had chemopreventive potential. The main aim of this study was to explore the chemopreventive properties of paddy waste against prostate disease. This study determines the antiproliferative activity of the paddy waste product in spontaneously hypertensive rats (SHRs).

**Methods:** Aqueous methanol extracts of paddy husk and straw were administered to SHRs for 17 weeks via drinking water, with no observed toxicity on dietary intake, body weight, liver, or kidney. The study used 18 male SHRs to model primary hypertension and 6 male Wistar Kyoto (WKY) rats as normotensive controls. The SHRs were divided into three groups: control (*n* = 6), paddy husk treated (*n* = 6, 15 mg/kg), and paddy straw treated (*n* = 6, 15 mg/kg), with treatment delivered in drinking water.

**Results:** It managed to reduce blood pressure (72.0 mmHg; *p*  < 0.01) and the size of the ventral prostate to around 0.05% (*p*  < 0.01). Histological analysis revealed antiproliferative signs such as a reduction in the number of acini (7.50; * p*  < 0.01), epithelial height (10.55 µm; *p*  < 0.01), and epithelial acinar area (18.17%; *p*  < 0.01). Aqueous methanol extracts have arrested the cell cycle by downregulating (*p*  < 0.01) proliferative marker, Ki-67, and proliferating cell nuclear antigen (PCNA). Prostate cell growth is arrested by downregulation of androgen receptor (AR) which inhibited AR mRNA transcription (RTPCR analysis) and induced cell cycle arrest at the S phase through p27 and cyclin E2 (western blot analysis).

**Conclusion:** In conclusion, paddy waste product especially husk is a better chemopreventive agent against prostate disease.

## 1. Introduction

Benign prostate hyperplasia (BPH) is a common noncancerous enlargement of the prostate gland in aging men [1]. While medical treatments like alpha-blockers and 5-alpha reductase inhibitors are commonly used, surgical options such as transurethral resection of the prostate (TURP) and laser surgery are available but often lead to complications like blood loss, erectile dysfunction, and urinary incontinence [2, 3]. Due to the high cost and side effects of these treatments, alternative therapies are increasingly sought after.

Natural products, including dietary and botanical sources, have shown promise as alternative treatments for prostate diseases. Among these, rice by-products like paddy husk and straw have emerged as potential chemopreventive agents. Paddy husk contains tocopherols, tocotrienols, phenolic acids, flavonoids, and momilactones, while paddy straw is rich in phenolics and flavonoids, all of which exhibit various bioactive effects relevant to BPH prevention. Tocopherols and tocotrienols, as forms of Vitamin E, possess antioxidant properties that reduce oxidative stress, a contributing factor to prostate enlargement [4–7]. Phenolic acids and flavonoids offer anti-inflammatory and proapoptotic effects by regulating key inflammatory pathways such as COX-2 and NF-*κ*B, while promoting apoptosis in abnormal prostate cells through caspase-3 activation [8]. Additionally, momilactones have antiangiogenic properties, inhibiting the formation of new blood vessels, which can otherwise contribute to excessive prostate growth [9]. The bioactive compounds found in paddy husk and straw may help regulate the biological changes such as oxidative stress, chronic inflammation, and excessive cell proliferation, that contribute to BPH, potentially reducing prostate enlargement and its related symptoms [10].

Since both paddy husk and straw have the potential to become chemopreventive agents, the aim of this study was to elucidate their effect on BPH by using an in vivo model, spontaneously hypertensive rats (SHRs). This work focuses on the biological changes associated with BPH in SHR and interventions with paddy waste products to prevent it. This work encompasses the evaluation of physical and metabolic parameters, histopathological analysis to investigate the morphological changes and distribution patterns of selected markers, and proteomic and genomic assessment to analyze the mechanism of paddy waste products on BPH.

## 2. Material and Methods

### 2.1. Preparation of Extracts

Ten grams of powdered paddy husk and straw were extracted through soxhlet extraction in 300 ml of 80% methanol, respectively, shaken at 10 × *g* for 3 days. After 3 days, the extracts were filtered using Whatman No. 1 filter paper. The supernatant was concentrated and lyophilized under reduced pressure using a rotary evaporator at 40°C to eliminate methanol. Finally, the remaining water content was removed by a freeze dryer to get dry extracts. The extracts were weighed and stored in −20°C refrigerator [11].

### 2.2. Animals

The strain of animals used were the SHRs and the control strain Wistar Kyoto (WKY) rats. Eighteen male young adult SHRs were used as a model of primary hypertension to study prostate hyperplasia and six male young adult WKY rats as normotensive models. SHRs were bought from BioLasco Taiwan Co., Ltd. (Taipei, Taiwan R.O.C.), while the WKY rats were bought from Animal Research and Service Center (ARASC), USM. All SHR and WKY rats were bought at the age of 4 and 14 weeks with initial weights of 128 ± 5 g and 246 ± 5 g, respectively. The Animal Ethics Committee USM approved the animal care and the experimental procedures [USM/Animal Ethics Approval/2014/(91)(561)]. The animals were housed in the Animal Research Complex (ARC), USM in individual stainless-steel cages in an environmentally controlled room kept at 22°C with a 12:12-h light: dark cycle and allowed to acclimatize for 2 weeks. Numerous pharmacological [12] and dietary studies [13] have used the SHR as a model of primary hypertension. The normotensive WKY is used as a control to the SHR since SHR as a strain was developed from outbred WKY rats [14]. The age of 10 weeks in the SHR is considered the early hypertensive stage, with a systolic BP at ~170 mmHg [15]. SHRs are expected to develop hypertension at the age of 7–15 weeks and their systolic blood pressure has been shown to plateau ~200 mmHg. Therefore, by 21 weeks of age, SHRs have developed full-blown hypertension [16].

### 2.3. Experimental Design and Sample Collection

Eighteen male young adult SHRs were divided into three groups: control (*n* = 6), rats without any treatment, paddy husk (*n* = 6), rats treated with paddy husk methanol extract (15 mg/kg) mixed in drinking water and paddy straw (*n* = 6), and rats treated with paddy straw methanol extract (15 mg/kg) mixed in drinking water. For the sample size calculation, the effect size was determined to be 0.8, indicating a substantial magnitude of the treatment's impact. Employing a standard alpha level of 0.05, the study's statistical significance threshold was established. With a power of 0.8, the study exhibited sufficient sensitivity to detect the hypothesized effects. The experimental design comprised four distinct groups, each consisting of six rats, ensuring an adequate sample size for robust statistical analyses and reliable inference. The dose of paddy waste extracts was based on studies done by Newaz et al. [17] and supported by work done by Huang and Ng [18]. The dosage calculation and stock solution preparation of extracts in drinking water were based on earlier work [19]. Another group consisted of WKY rats (*n* = 6) which served as a normotensive model. Food and water consumption were given ad libitum and measured daily together with the weight of each rat. The blood pressure level of rats was checked every 4 weeks. After 17 weeks of treatment, the rats were euthanized by using ketamine–xylazine and the target tissues: ventral prostate, liver, and kidney were excised and weighed. Target tissue used for the histology, immunohistochemistry, western blot, and real-time polymerase chain reaction (PCR) analysis.

### 2.4. Tissue Processing and Histopathology

Biopsies were embedded in paraffin using an automated tissue processor (Leica TP1050, Leica Instruments GmbH, Nussloch, Germany) and cut into 5 μm sections that were arranged onto microscope slides. To remove paraffin from the tissue surface, tissue sections were incubated in xylene for 2 min (2x), rehydrated 2 min each in a serial grade of absolute (2x), 95%, 80%, 70%, and 50% ethanol, and finally washed in running tap water for 3 min. The staining was performed by placing deparaffinized tissue sections in Harris hematoxylin (Electron Microscopy Science, USA) for 3 min before rinsing thoroughly in running tap water until slides become clearer. Tissue sections were dipped a few times in 0.5% acid alcohol and 0.3% ammonia until sections appeared bright blue. The sections were counterstained with Eosin (Electron Microscopy Science, USA) for 1 min. Between each step, slides were cleaned under running tap water. The tissue is then dehydrated in serial grade alcohols: 95% alcohol (1 min), absolute alcohol (2 × 2 min), and immersed in xylene (3 × 2 min) before mounting in dibutylphthalate polystyrene xylene (DPX) mountant (Electron Microscopy Science, USA) after slides dried. Further analysis involving histomorphometric analysis: number of acini, percentage of relative epithelial to acinar area, and the average height of epithelial of ventral prostate were measured using Image_J 1.41 program [20].

### 2.5. Immunohistochemistry Analysis

Tissue sections were incubated in xylene for 2 min (2x) to remove paraffin from the tissue surface. After that, tissue sections were rehydrated for 2 min each in a serial grade of absolute (2x), 95%, 80%, 70%, and 50% ethanol and finally washed in running tap water for 3 min. Sections were incubated in 3% H_2_O_2_ solution in methanol for 10 min to block endogenous peroxidase activity before rinsing with phosphate-buffered saline (PBS) two times, 5 min each. A hundred milliliters of blocking buffer were added to the sections of slides and incubated at room temperature for 1 h. After draining off the blocking buffer, 100 μl of diluted primary antibody (Rabbit Polyclonal Androgen Receptor [AR] from Abcam, USA, or Ki67 Antibody from Thermo Fischer Scientific, USA) was added to the sections on the slides and incubated at room temperature for 1 h. After that, 100 μl of goat antirabbit immunoglobulin G (IgG) (HRP [horseradish peroxidase]) was added to the sections and incubated at room temperature for 30 min. Between each step, slides were washed with PBS two times, 5 min each. In total, 100 μl of signal stain 3,3′-diaminobenzidine (DAB) substrate kit (Cell Signaling Technology, USA) solution was applied to the sections for 5 min and washed with PBS three times, 2 min each. The slides were counterstained with Harris hematoxylin (Electron Microscopy Science, USA) for 2 min and rinsed in running water for 10 min. The tissue slides were dehydrated through four times of alcohol (95%, 95%, 100%, and 100%), 5 min each. Tissue slides cleared three times of xylene and mounted with DPX mountant (Electron Microscopy Science, USA) for observation under microscopy. Further analysis involving histochemistry analysis of the ventral prostate was done using the Image_J 1.41 program (NIH, Bethesda, USA) [21].

#### 2.5.1. Western Blot

Cell extracts containing 30 µg of protein were mixed with an equal volume of sample buffer containing 12 mM Tris-HCl, pH 6.8, 6% SDS, 20% glycerol, 10% B-mercaptoethanol, and 0.03% bromophenol blue. Protein samples were boiled for 5 min and electrophoresed under reducing conditions using from 8% to 12% resolving gel. Separated proteins were electrophoretically transferred to a nitrocellulose membrane and blocked with a blocking reagent for 1 h at room temperature. The membrane was washed three times (15 min each) with washing buffer (0.1% [v/v] Tween 20, 1x tris buffer saline [TBS]). Immunodetection was performed overnight with gentle shaking at 4°C in 1x TBS, 5% (w/w) bovine serum albumin and 0.1% Tween 20 using primary antibodies that were diluted according to the manufacturer's instructions. Primary antibodies used in this experiment were rabbit polyclonal AR (Abcam, USA), p27 Kip1 antibody (Cell Signaling Technology, USA), cyclin E2 antibody (Cell Signaling Technology, USA), and *β*-actin antibody (Cell Signaling Technology, USA). The membrane then was washed three times with washing buffer and incubated with a 1:3000 dilution of antimouse or antirabbit alkaline phosphatase-conjugated secondary antibody for 1 h. Bound proteins were detected using the Amersham ECL Kit and then exposed to Hyperfilm (Amersham, Little Chalfont, United Kingdom) in a dark room. The films were then inserted into an automatic film developer's machine (Fuji, Tokyo, Japan).

### 2.6. Real-Time PCR

Total ribonucleic acid was extracted from tissues using Trizol reagent (Invitrogen, Carlsbad, CA) according to the manufacturer's protocol. All RNA samples were rid of contaminating DNA by using DNA-free reagents (Invitrogen, Carlsbad, CA) according to the manufacturer's protocol. One microgram of RNA was reversed transcripted with Superscript III (Invitrogen). The real-time PCR amplification was performed using 2 μl of cDNA, specific primers for each gene, and SyBR Green reagent (Invitrogen), in a final volume of 20 μl. The reactions were performed in triplicate. The primer sequences used are AR: forward primer: 5′-TTG TGA ACA GAG TCC CCT AT-3′, reverse primer: 5′-TTC TGG GAT GGG TCC TCA GT-3′. Housekeeping gene GAPDH: forward primer: 5′-GCG AGA TCC CGT CAA GAT CA-3′, reverse primer: 5′-CCA CAG TCT TCT GAG TGG CAG-3′.

### 2.7. Statistical and Data Analysis

Statistical analysis was conducted using SPSS 22.0 for Windows (SPSS Inc., Chicago, IL). For each experiment, three independent experiments (*n* = 3) were conducted. Data were represented in mean ± SD manner of triplicates. One-way analysis of variance (ANOVA) with post hoc Tukey test was used to evaluate the differences between treated samples and untreated control. *p* values less than 0.5 and 0.01 were considered statistically significant.

## 3. Results

The effect of paddy waste aqueous methanol extracts on body weight, food, and water intake.

SHRs were weighed each week during the 17 weeks of dietary intervention. There were no significant differences between the final mean body weights, food, and water intake of each group analyzed by one-way ANOVA with the Tukey test.

Blood pressure in SHR and WKY rats after treatment with paddy waste aqueous methanol extracts.

Throughout the study, it was found that there was a significant difference between blood pressure in control animals versus those fed paddy waste aqueous methanol extracts in drinking water.  Table 1 indicates that paddy waste products; husk and straw managed to lower the systolic blood pressure in SHR rats after 16 weeks of treatment in drinking water. The normotensive rat group, WKY, showed constant blood pressure readings during 16 weeks of treatment with systolic blood pressure averaging 130.2 ± 8.8 mmHg (*p*  < 0.05) during the first 4 weeks and 135.0 ± 5.1 mmHg (*p*  < 0.05) after 16 weeks of treatment.

**Figure 1:**
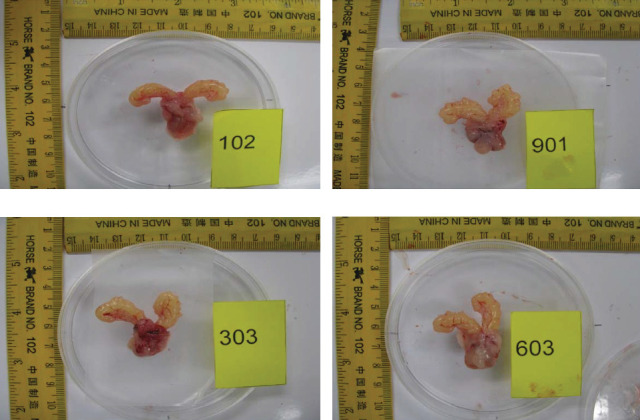
Photograph showing the gross features and the size of the prostate among studied groups: (A) control SHR group, (B) normotensive WKY group, (C) paddy husk group, and (D) paddy straw group. After treatment, paddy husk (C) and straw (D) managed to decrease the volume of organ/body weight from 0.12% in control to 0.07% (*p*  < 0.05) (husk) and 0.08% (*p*  < 0.05) (straw). SHR, spontaneously hypertensive rat; WKY, Wistar Kyoto.

Relative organ weight analysis: Analysis of relative organ weight is an important endpoint for the identification of potentially harmful effects of paddy waste products. The analysis was done in three types of organs, ventral prostate, liver, and kidney, as shown in Table 2. Relative ventral prostate weight analysis showed that there was a significant decrease in percentage compared to control SHR. This result indicated that treatment of paddy husk and straw managed to decrease the volume of organ/body weight from 0.12% in control to 0.07% (*p*  < 0.05) and 0.08% (*p*  < 0.05) in paddy husk and straw, respectively. Analysis of the other two organs: the liver and kidney showed an insignificant decrease of relative organ weight that indicated no presence of harmful substances in the paddy waste product that potentially induced toxicity effect. The gross appearance of the prostate gland between the groups is reflected in Figure 1.

**Figure 2:**
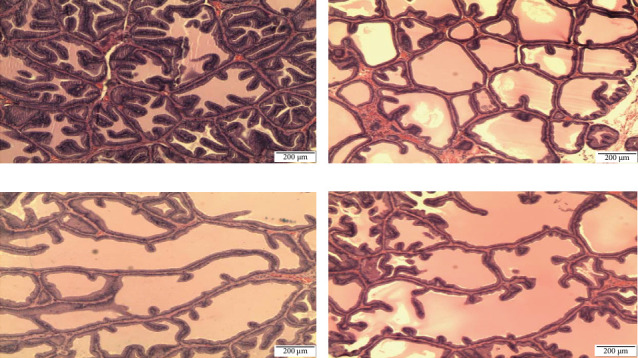
Photomicrograph showing the differences in the number of acini in the ventral prostate lobe of (A) control SHR group, (B) normotensive WKY group, (C) paddy husk group, and (D) paddy straw group. The magnification of all photomicrographs is 40x. Figure 1 shows the differences in the number of acini in each group where the average number of acini in the control group was 19.00 ± 2.28 and after being treated with paddy waste, the average number of acini significantly decreased in both treated groups; the paddy husk group (11.50 ± 2.59) and paddy straw group (12.00 ± 2.83). SHR, spontaneously hypertensive rat; WKY, Wistar Kyoto.

**Figure 3:**
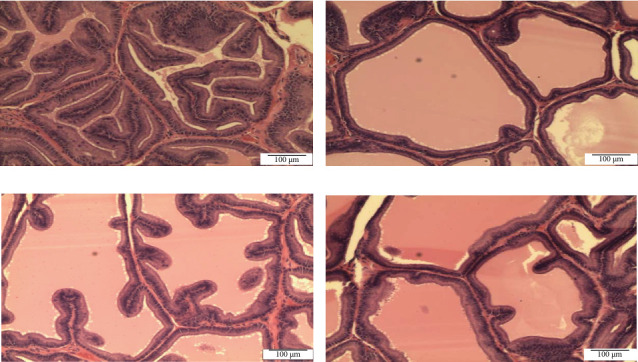
Photomicrograph showing the differences of epithelial and luminal area in ventral prostate lobe of (A) control SHR group, (B) normotensive WKY group, (C) paddy husk group, and (D) paddy straw group. Magnification of all photomicrographs is 100x. Figure 2 shows the alterations of epithelial and luminal areas in each group. The relative epithelial area to acinar area in the control group (60.62% ± 3.32) decreased significantly after treatment with paddy waste product especially in the paddy husk group (42.45% ± 5.36) and paddy straw group (51.66% ± 6.24).

**Figure 4:**
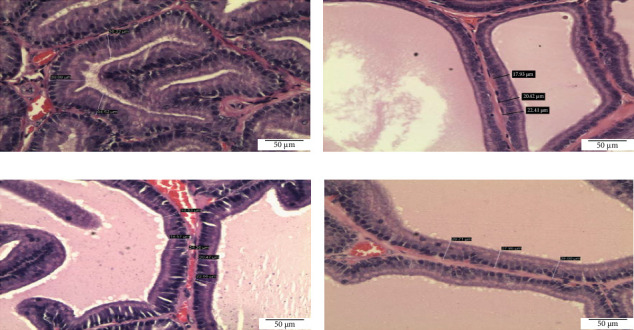
Photomicrograph showing the differences of epithelial height in ventral prostate lobe of (A) control SHR group, (B) normotensive WKY group, (C) paddy husk group, and (D) paddy straw group. Magnification of all photomicrographs is 200x. Figure 3 shows the differences in epithelial height in each group. The average epithelial height of the control group was 35.70 μm ± 2.65 and after treatment with paddy waste product, the average epithelial height decreased significantly to 20.15 μm ± 2.12 (paddy husk group) and 25.58 μm ± 3.36 (paddy straw group). SHR, spontaneously hypertensive rat; WKY, Wistar Kyoto.

Histomorphometric analysis in the ventral prostate of SHR rats: Histomorphometric analysis of ventral prostate of each group, (a) control untreated SHR, (b) WKY normotensive group, (c) paddy husk group, and (d) paddy straw group, was divided into three parameters, as shown in Table 3; average number of acini in each group (Figure 2), percentage of relative epithelial area to acinar area (Figure 3), and average of epithelial height in each group (Figure 4). The WKY group was considered as a normal positive control for each histomorphometric analysis. Treatment of paddy waste product does not cause significant alteration to the histologic structures of dorsolateral and seminal vesicles. This result showed that paddy waste product caused alteration specifically on the ventral prostate. Based on toxicology studies, paddy waste products showed no significant changes in the morphology of the liver and kidney structure after 16 weeks of treatment. Figure 2 shows the differences in the number of acini in each group where the average number of acini in the control group was 19.00 ± 2.28 and after being treated with paddy waste, the average number of acini significantly decreased in both treated groups; the paddy husk group (11.50 ± 2.59) and paddy straw group (12.00 ± 2.83). Figure 3 shows the alterations of epithelial and luminal areas in each group. The relative epithelial area to acinar area in the control group (60.62% ± 3.32) decreased significantly after treatment with paddy waste product especially in the paddy husk group (42.45% ± 5.36) and paddy straw group (51.66% ± 6.24). Figure 4 shows the differences in epithelial height in each group. The average epithelial height of the control group was 35.70 μm ± 2.65 and after treatment with paddy waste product, the average epithelial height decreased significantly to 20.15 μm ± 2.12 (paddy husk group) and 25.58 μm ± 3.36 (paddy straw group).

**Figure 5:**
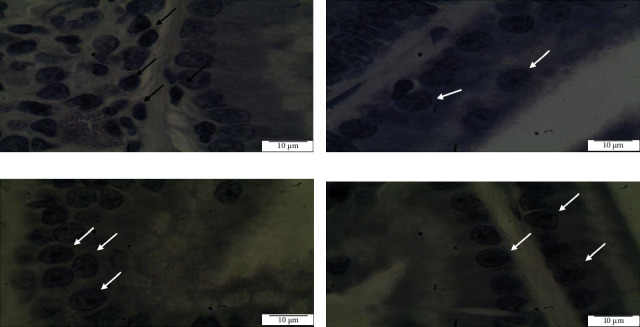
Immunohistochemical staining for androgen receptor (AR) in ventral prostate of control SHR (A), WKY (B), paddy husk methanol extract group (C), and paddy straw methanol extract group (D). (A) AR expression was found higher in cell nucleus (black arrows); (B) AR staining less intense in ventral prostate of WKY tissue; and (C, D) downregulated AR expression was found in cell nucleus (white arrows). Magnification ×1000 for all images. SHR, spontaneously hypertensive rat; WKY, Wistar Kyoto.

**Figure 6:**
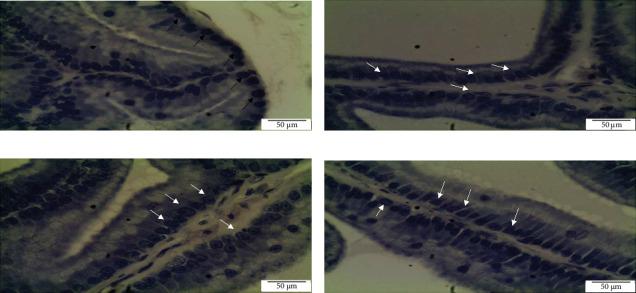
Immunohistochemical staining for Ki-67 in ventral prostate of control SHR (A), WKY (B), paddy husk methanol extract (C), and paddy straw methanol extract (D). (A) Ki-67 expression was found in cell nucleus of ventral prostate tissue and the arrow indicates the positive area in the tissues (black arrows). (B) Ki-67 staining less intense in ventral prostate of WKY tissue; (C, D) downregulated Ki-67 expression was found in cell nucleus (white arrows). Magnification ×200 for all images. SHR, spontaneously hypertensive rat; WKY, Wistar Kyoto.

AR and Ki 67 expression in the ventral prostate of SHR and WKY rats: The specificity and sensitivity of the anti-AR and Ki-67 antibodies used to recognize its antigen were confirmed by the staining in the positive reaction of all the prostate tissue sections (Figures 5 and 6, respectively). WKY group acted as a normal positive control. AR immunoreactivity was almost exclusively nuclear and was observed in the glandular epithelial cells. The mean number of stained positive cells (black arrow) was significantly higher in control SHR than in paddy waste husk and straw-treated prostate tissues (white arrow). The percentage of AR labeling index was higher in control SHR (16.45%) reflecting on the accumulation of AR protein (dark stain) in the nucleus of epithelial cells. The labeling index was significantly lower in paddy waste product treated samples, as shown in Table 4; paddy husk (5.46%) and paddy straw (7.23%). This result showed that paddy waste product managed to downregulate significantly the AR expression in the ventral prostate of SHR. Ki-67 expression was mainly localized in cell nuclei and the labeling index of control SHR was 17.76% and significantly decreased in treated samples; paddy husk (6.32%) and paddy straw (8.13%). These results showed that paddy waste product managed to downregulate significantly the expression of proliferation marker, Ki-67 in the ventral prostate of SHR.

Effect of paddy waste extracts on protein expression in ventral prostate of SHR rats: There were two types of methanol extracts (husk and straw) analyzed to determine the expression of markers involved in proliferation and androgen-dependent cell cycle, as shown in Table 5. Proliferating cell nuclear antigen (PCNA) is a proliferative marker that showed a significant reduction in expression after treatment compared to control. This indicated that paddy waste methanol extracts managed to reduce the proliferation of prostate cells. The highest increase of fold difference is p27^kip1^ while all other markers showed a decrease in fold difference. Paddy husk showed better fold differences than paddy straw except for PCNA. Protein markers that were involved in androgen-dependent cell cycle had been targeted with reduction of AR expression causing significant elevation of p27^kip1^ that subsequently inactivated the expression of cyclin E2 which caused deactivation of S-phase in the cell cycle. The *β*-actin protein was used as loading control of western blot analysis. When compared between both types of paddy waste products, paddy husk methanol extract showed a better reaction with elevation (PCNA, cyclin E2, and AR) and reduction (p27^kip1^) folds greater than paddy straw methanol extract.

**Figure 7:**
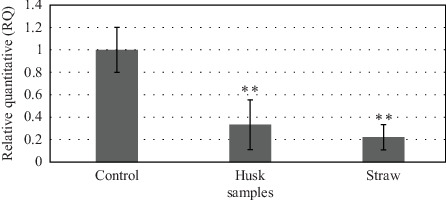
Verification of expression differences in candidate gene (androgen receptor) in ventral prostate of SHR using relative quantification (RQ). Control group indicates gene expression verification of untreated SHR ventral prostate while husk and straw groups indicate gene expression verification of SHR ventral prostate after being treated with paddy husk and straw aqueous methanol extracts. The androgen receptor gene showed a lower expression level after being treated with paddy waste product. Values were represented in ±SD manner of triplicates. *⁣*^*∗*^*p*  < 0.05, *⁣*^*∗∗*^*p*  < 0.01 as compared to control untreated by using one-way ANOVA with post hoc Tukey test. ANOVA, analysis of variance; SHR, spontaneously hypertensive rat.

Effect of paddy waste extracts on gene expression in the ventral prostate of SHR rats: The gene expression analysis was done to analyze the AR gene expression of the ventral prostate of SHR after being treated with paddy waste product against untreated control, as shown in Figure 7. The mRNA expression levels of ARs were reduced as compared to normal prostate. Relative quantitative value was reduced to 0.33 for the paddy husk methanol extract treated sample and 0.22 for the paddy straw methanol extract treated sample.

### 3.1. Statistical Analysis

The normality assumption was fulfilled by using Shapiro–Wilk test with *p*  > 0.05. Then, parametric analysis was conducted using SPSS 29.0 for Windows (SPSS Inc., Chicago, IL). For each experiment, three independent experiments (*n* = 3) were conducted. Data were represented in mean ± SD manner of triplicates. One-way ANOVA with post hoc Tukey test was used to evaluate the differences between treated samples and untreated control. *p* value less than 0.5 and 0.01 was considered statistically significant.

## 4. Discussion

The present study showed, for the first time to our knowledge, antiproliferative effects of paddy waste product on rat prostate hyperplasia growth through AR downregulation in a SHR model. Good in vivo study plays an important role in bridging the in vitro studies and clinical trials. This in vivo study is a crucial step in proving the credential of paddy waste products as good chemopreventive agents before recommending them for human clinical trials. The body weight analysis assessed for 17 weeks of observation showed that paddy waste aqueous methanol extracts did not induce significant changes in final body weights between the control and treated animal groups. The animal's final body weight is an important factor in evaluating the toxicity of crude extracts [22]. Any weight loss that occurs exceeds 10% of the initial body weight indicating the adverse effects of toxic extracts [23]. There were no significant changes in water consumption and food intake in the treated groups compared to the control group. These dietary intake parameters are important to evaluate the safety of paddy waste products against any toxicity effects [24].

A SHR is a genetically hypertensive rat that develops a similar condition to BPH [25]. SHR develops an early hypertensive stage by the age of 10 weeks [14] and the systolic blood pressure reaches 200 mmHg by age 15 weeks [15]. At age 15 weeks, SHR develops age-associated BPH where there are hyperplasia-related morphological abnormalities that can be seen in the ventral prostate [25–27]. All these reports proved that the ventral prostate of the SHR can be crucial for human BPH research. Previous work showed that WKY rats were used as normotensive control rats since there was no significant difference in androgen hormone levels between SHR and WKY rats [28]. In the present study, evidence presented showed that there were strong significant differences (*p*  < 0.01) between the paddy waste product aqueous methanol extracts treated group against a control group. Paddy waste product aqueous methanol managed to lower the systolic blood pressure in SHR rats after 16 weeks of treatment. Since hypertension is a risk factor for BPH, lowering systolic blood pressure is crucial for BPH treatment [29]. Previous works proved that targeting systolic blood pressure is an important step in treating BPH. Ucar et al. [30] aimed to assess the effects of silodosin, a selective adrenergic alpha-1 antagonist, on arterial stiffness in patients with BPH. Silodosin significantly reduced International Prostate Symptom Scores. Meanwhile, Bukowska et al. [31] gave evidence that doxazosin, an alpha blocker, prevented the development of hypertension-induced changes in SHR. The relative ventral prostate weight is one of the important markers of BPH development. In the present study, the animals treated with paddy waste aqueous methanol extracts showed a significant reduction compared to the control group (*p*  < 0.05). These results indicate that paddy waste aqueous methanol extracts attenuated the ventral prostate enlargement. These results showed that paddy waste aqueous methanol extracts can be a good antiproliferative agent for BPH. This claim is supported by earlier work where lauric acid and myristic acid were isolated from the plant used to treat BPH and managed to reduce the relative organ weight of the ventral prostate [32]. The relative organ weight is important to diagnosing the organ injury or whether affected by the metabolic reaction of toxic extracts. The relative weights of isolated vital organs, liver and kidney, showed that there was no significant difference between the treated and the control groups, showing that paddy waste aqueous methanol extracts were nontoxic in these vital organs [23]. In the present study, the animals treated with paddy waste aqueous methanol extracts showed significant differences histologically compared to the control group. The number of acini in treated samples is lower than in the control group. This is supported by earlier work where the anticancer and antiaging effects of rapamycin, which delay cancer by slowing aging and targeting precancerous cells studied. Rapamycin managed to abrogate the enlargement of the prostate by normalizing the tissue architecture and reducing the number of acini [33]. The percentage of the relative epithelial acinar area significantly decreased in the animals treated with paddy waste aqueous methanol extracts compared to the control group. This finding is similar to previous work by Borovskaya et al. [34]. They have proved that the drug Tertapeptide Lys-Glu-Asp-Pro managed to decrease the epithelial acini area and was considered for medication for the therapy of BPH in experimental rats [34]. Paddy waste aqueous methanol treatment has reduced significantly the epithelial height of the SHR ventral prostate hyperplasia compared to the control group. This result gives a similar effect of androgen deprivation of prostate hyperplasia and is supported by earlier work [35]. Two proliferative markers, Ki-67 and PCNA, were chosen to determine the antiproliferative effect of paddy waste aqueous methanol treatment on the ventral prostate of SHR. Ki-67 antigen can be used as a useful index for cell proliferation because it was expressed in proliferative cells throughout cell cycle phases. Meanwhile, PCNA is an acidic nuclear protein that is expressed specifically in phase S of the cell [36]. Immunohistochemistry analysis revealed that paddy waste aqueous methanol treatment managed to significantly reduce the expression of Ki-67 on treated SHR ventral prostate hyperplasia compared to the control group. Ki-67 is a mitotic activity indicator that is normally detected in the late G1 phase. Downregulation of Ki-67 indicates low cell proliferation and mitotic activity [37]. Meanwhile, western blot analysis showed that paddy waste aqueous methanol treatment managed to reduce significantly the expression of PCNA compared to the control group. PCNA is a protein that is involved in the proliferation of cells and expressed in proliferating cell nuclei. This protein is normally at peak level in the late G1 and S phases of proliferating cells [38].

AR plays an important role in promoting the proliferation of prostate epithelial cells. Valentine et al. [39] found that the addition of the synthetic androgen mibolerone increased AR expression level and enhanced the prostate cancer cell line. Thus, the importance of understanding the role of ARs in BPH glandular epithelial cells is crucial for BPH prevention and treatment. Immunohistochemistry analysis revealed that AR expression was downregulated after treatment with paddy waste aqueous methanol. This result is supported by earlier work by Kim et al. [40]. They managed to suppress the expression of AR when the prostate hyperplasia of Sprague Dawley was treated with *Cinnamomum Cassia* and *Rosa Laevigata* mixture [40]. They use cinnamon cortex to suppress 5AR (3-oxo-5*α*-steroid 4-dehydrogenases) an enzyme involved in steroid metabolism. Since 5AR is important for prostatic hyperplasia development and is able to downregulate the level of AR, these results concluded that the cinnamon cortex can be a potential pharmaceutical therapy for BPH treatment.

For gene expression analysis, the AR expression in paddy waste product aqueous methanol treated sample compared to control ventral prostate SHR. The expression of AR mRNA in the treated sample is about threefold lower compared to control ventral prostate SHR. This finding is consistent with the literature. According to Kitajima and Takahashi, androgens signaling through ARs is important for the development of BPH and AR gene level in BPH is always higher than the normal prostate tissues [41]. In the present study, our western blot analysis revealed that treatment of paddy waste aqueous methanol activated AR-dependent cell cycle pathway. The result involves three proteins involved in the AR-dependent cell cycle pathway; p27kip1, cyclin E2, and AR. The expression of p27kip1 was increased while cyclin E2 and AR were downregulated after treatment. There is one possible mechanism that can be proposed about the effect of paddy waste aqueous methanol extracts on AR-induced proliferation of the ventral prostate. Since the expression of 5*α*-reductase II in BPH tissues is higher, paddy waste aqueous methanol extracts inhibit 5*α*-reductase II which blocks the conversion of testosterone into dihydrotestosterone. This will lead to a reduction of AR that is involved in the enlargement of the prostate [42]. AR deprivation will elevate the level of p27kip1, a tumor suppressor protein, which in turn will suppress cyclin E2/cdk expression. Although the complete mechanism is not fully understood this eventually will cause cell cycle arrest during the transition from G1 to S phase [43]. Since paddy waste product is considered as novel compound, more possible mechanisms should be explored. There are a few analyses that can be done in the future to strengthen the reputation of paddy waste products as good chemopreventive agent. Phytochemical study on both water and aqueous methanol extracts can be done to find the types of phytochemicals found in both extracts. Paddy waste product caused apoptosis on prostate cancer cells through caspase 3-mediated intrinsic apoptotic pathway and antiproliferative effect by downregulating androgen-dependent cell cycle on SHR rat ventral prostate hyperplasia. The two-dimensional electrophoresis system can be used in future research for complete quantitative analysis of protein expression involved in both mechanisms.

## 5. Conclusion

This study investigates the potential of paddy waste (husk and straw extracts) as chemopreventive agents against prostate cancer and BPH. In vitro studies revealed that paddy husk aqueous methanol extract effectively inhibited androgen-independent prostate cancer (DU145) cell growth through apoptosis, with caspase 3 activation and apoptotic DNA fragmentation. In vivo studies showed that paddy waste aqueous methanol extracts, particularly paddy husk, reduced systolic blood pressure and had antiproliferative effects on SHR ventral prostate without toxicity. Protein and gene expression analysis confirmed that these extracts regulate key pathways involved in BPH. Thus, paddy husk aqueous methanol is a promising candidate for prostate cancer and BPH prevention.

## Figures and Tables

**Table 1:** Systolic blood pressure of SHR and WKY rats after 16 weeks of treatment.

Samples	4 weeks (mmHg)	8 weeks (mmHg)	12 weeks (mmHg)	16 weeks (mmHg)
Control SHR	186.0 ± 7.5	191.0 ± 7.5	215.8 ± 12.5	229.0 ± 8.9
Husk SHR	170.0 ± 7.2^*∗*^	167.7 ± 7.6^*∗∗*^	160.0 ± 6.9^*∗∗*^	157.8 ± 3.4^*∗∗*^
Straw SHR	172.5 ± 6.8^*∗*^	173.2 ± 5.3^*∗∗*^	169.0 ± 4.5^*∗∗*^	165.3 ± 4.9^*∗∗*^
WKY	130.2 ± 8.8^*∗∗*^	131.2 ± 5.4^*∗∗*^	133.3 ± 7.0^*∗∗*^	135.0 ± 5.1^*∗∗*^

Abbreviations: ANOVA, analysis of variance; SHR, spontaneously hypertensive rat; WKY, Wistar Kyoto.

*⁣* ^*∗*^ *p*  < 0.05, *⁣*^*∗∗*^*p*  < 0.01 as compared to control untreated by using one-way ANOVA with post hoc Tukey test.

**Table 2:** Relative organ weight of ventral prostate, liver, and kidney of SHR rats.

Group	Number of rats	Relative organ weight (%)		
		Ventral prostate	Liver	Kidney
Control	4	0.12 ± 0.02	4.59 ± 0.13	0.83 ± 0.03
Husk	4	0.07 ± 0.01^*∗*^	4.54 ± 0.17	0.78 ± 0.04
Straw	4	0.08 ± 0.02^*∗*^	4.52 ± 0.10	0.74 ± 0.07

Abbreviations: ANOVA, analysis of variance; SHR, spontaneously hypertensive rat.

*⁣* ^*∗*^ *p*  < 0.05 as compared to control untreated by using one-way ANOVA with post hoc Tukey test.

**Table 3:** Histomorphometric analysis of ventral prostate of treated and control SHR rats.

Samples	Average number of acini in section	Relative epithelial area to acinar area (%)	Average of epithelial height (μm)
Control	19.00 ± 2.28	60.62 ± 3.32	35.70 ± 2.65
Husk	11.50 ± 2.59^*∗∗*^	42.45 ± 5.36^*∗∗*^	20.15 ± 2.12^*∗∗*^
Straw	12.00 ± 2.83^*∗∗*^	51.66 ± 6.24^*∗∗*^	25.58 ± 3.36^*∗*^
WKY	11.50 ± 4.76^*∗∗*^	38.02 ± 6.47^*∗∗*^	20.25 ± 2.24^*∗∗*^

Abbreviations: ANOVA, analysis of variance; SHR, spontaneously hypertensive rat; WKY, Wistar Kyoto.

*⁣* ^*∗*^ *p*  < 0.05, *⁣*^*∗∗*^*p*  < 0.01 as compared to control untreated by using one-way ANOVA with post hoc Tukey test.

**Table 4:** Effects of paddy waste product on the androgen receptor and Ki-67 labeling index in ventral prostate of SHR and WKY.

Samples	Androgen receptor (AR) labeling index (%)	Ki-67 labeling index (%)
Control SHR	16.45 ± 5.45	17.76 ± 4.37
WKY	10.67 ± 2.14	12.45 ± 3.98
Paddy husk	5.46 ± 2.17^*∗∗*^	6.32 ± 2.54^*∗∗*^
Paddy straw	7.23 ± 2.13^*∗∗*^	8.13 ± 2.87^*∗∗*^

Abbreviations: ANOVA, analysis of variance; SHR, spontaneously hypertensive rat; WKY, Wistar Kyoto.

*⁣* ^*∗*^ *p*  < 0.05, *⁣*^*∗∗*^*p*  < 0.01 as compared to control untreated by using one-way ANOVA with post hoc Tukey test.

**Table 5:** This table shows the result of the image (band) after analyses with Image J software to evaluate the differences in band intensity which ensures the significance of the result.

Target protein	Control	Husk	Straw	kDa
PCNA	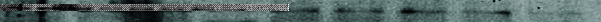			36
	1.0	0.7	0.6	
Cyclin E2	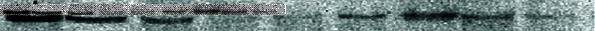			48
	1.0	0.6	0.8	
p27_kip1_	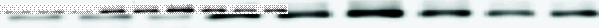			27
	1.0	2.8	2.0	
Androgen receptor	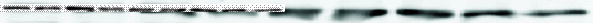			100
	1.0	0.7	0.8	
*β*-Actin	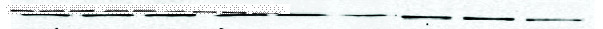			45
	1.0	1.0	1.0	

## Data Availability

The data that support the findings of this study are available from the corresponding author upon request.

## Nomenclature

COX-2: Cyclooxygenase-2
NF-κB: Nuclear factor kappa-light-chain-enhancer of activated B cells
H_2_O_2_: Hydrogen peroxide
PBS: Phosphate-buffered saline
Ki67: Marker of proliferation Ki-67
IgG: Immunoglobulin G
HRP: Horseradish peroxidase
DAB: 3,3'-Diaminobenzidine
DPX: Dibutylphthalate polystyrene xylene
HCl: Hydrochloric acid
ECL: Enhanced chemiluminescence
GAPDH: Glyceraldehyde-3-phosphate dehydrogenase.
